# Responsive Feeding Practices Among Caregivers of Children Aged 6-35 Months in China: Descriptive Study Involving Survey and Video Observation Methods

**DOI:** 10.2196/78028

**Published:** 2026-02-26

**Authors:** Dongmei Liu, Yujie Wen, Meijing An, Nan Wu, Xiaojing Ren, Xiyao Liu, Jie Huang, Qianling Zhou

**Affiliations:** 1 Department of Maternal and Child Health, School of Public Health, Peking University, No. 38 Xueyuan Road, Haidian District, Beijing, 100191, China, 86 13811628250; 2 Jinan Maternity and Child Care Hospital Affiliated to Shandong First Medical University, Jinan, Shandong Province, China; 3 Loudi Town Hospital of Luancheng District, Shijiazhuang, Hebei Province, China; 4 School of Public Health and Emergency Management, Southern University of Science and Technology, Shenzhen, Guangdong Province, China

**Keywords:** responsive feeding, survey, video observation, infant and toddler, factors

## Abstract

**Background:**

Responsive feeding is an integral component of nurturing care under the umbrella of early childhood development and has been recommended as an optimal feeding practice globally.

**Objective:**

This study was conducted to explore responsive and nonresponsive feeding practices among caregivers of children aged 6‐35 months in China. Factors influencing responsive/nonresponsive feeding practices were further explored.

**Methods:**

This study used a combination of survey and video observation approaches and was conducted in Hebei Province from August to October 2020. A cross-sectional survey (n=409) was conducted to measure caregivers’ responsive/nonresponsive feeding practices using a prevalidated scale (5-point Likert scale). The overall and individual dimension scores were calculated. Multiple linear regression was performed to explore the demographic factors associated with responsive/nonresponsive feeding practices. Video observation was conducted among 42 caregiver-child pairs to record the dining episodes of main meals for a day at participants’ homes. Videos taken were coded, and the feeding practices were extracted. The occurrence of each feeding practice was calculated. The results from the 2 methods were confirmatory and complementary to each other.

**Results:**

Caregivers adopted responsive feeding more frequently than nonresponsive feeding, with a median overall responsive feeding score of 3.52 (IQR 3.36-3.76) in the survey and a higher occurrence in video observation (responsive vs nonresponsive feeding: 75.6%‐97.6% vs 0%-46.3%). No significant differences in feeding practices were found across breakfast, lunch, and dinner (all *P*>.05). Nonparental caregivers (β=0.13, 95% CI 0.05-0.21) and those with a household monthly income of >5000 RMB (>US $721; β=0.07, 95% CI 0.01-0.14) had a higher score for overall responsive feeding, while unemployed caregivers (β=−0.07, 95% CI −0.13 to −0.01) had a lower score.

**Conclusions:**

Caregivers appear to be more likely to use responsive feeding practices than nonresponsive feeding practices. Interventions to promote responsive feeding should target parental, unemployed, and low-income caregivers. The findings of this study might serve as a reference for the comprehensive assessment of responsive feeding practices.

## Introduction

Nurturing care is an essential element in the promotion of early childhood development [1]. In 2018, the World Health Organization (WHO) and other international organizations jointly proposed 5 core elements of nurturing care [2]. One of the elements is responsive parenting, which requires caregivers to discern children’s needs from their movements, sounds, expressions, and oral requests in daily life, and to provide timely and appropriate responses [3]. Responsive feeding refers to the positive interaction between caregivers and infants during the feeding process, where caregivers provide timely and appropriate responses to signals of hunger and satiety in infants and young children [3]. Responsive feeding has been incorporated into the infant and young child feeding guidelines by the WHO [4]. On the contrary, nonresponsive feeding should be avoided. As described by our research team, responsive feeding practices include the practices of responsiveness to cues, modeling, active communication and interaction, and creating a good meal environment, while nonresponsive feeding practices include controlling, pressure to eat, the use of food as a reward, and emotional feeding [5,6].

Previous studies about responsive/nonresponsive feeding have mainly been conducted in North America, Europe, and Australia [7,8]. A study in the United States demonstrated that the average frequencies of responsive feeding practices (eg, modeling and reasoning) at breakfast, lunch, and dinner were 9.2, 9.7, and 15.4 times, respectively, while the frequencies of nonresponsive feeding practices (eg, coercive-controlling prompts) at the same points were 2.9, 3.0, and 5.8 times, respectively [7]. A study in Australia revealed that the scores for nonresponsive feeding among caregivers in Australia were relatively low, with a mean score of 1.86 (SD 0.99) for the use of food as a reward and 1.99 (SD 0.64) for emotional feeding among caregivers of 2-year-old children [8]. Moreover, some studies have investigated responsive/nonresponsive feeding in China, with the majority (16/20) conducted in southern China. Chinese studies examined the practices of pressure to eat, the use of food as a reward, and active communication and interaction, while there is a paucity of reports on controlling, responsiveness to cues, modeling, and creating a good meal environment [9].

Responsive/nonresponsive feeding practices are typically assessed by survey or observation methods, each with distinct advantages and limitations. The survey method, which has been adopted by most studies [10], allows for large-scale data collection. However, existing tools used by surveys are unable to cover all dimensions of feeding practices [11]. In the Chinese context, certain practices (eg, controlling and responsiveness to cues) are still underreported [9]. Most observation studies used videos to record the process of feeding [12]. Home is the natural environment where children dine. Thus, taking videos to record the feeding episodes at home is feasible and can reflect real-life feeding practices. However, the observation method is only suitable for studies with a small sample size due to its high cost and respondent burden. Combining survey and observation methods in a single study can obtain rich information and explore the topic in-depth [13-15]. The survey provides quantitative breadth, while the observation yields qualitative depth, and they together offer a holistic understanding of feeding practices. For example, a study in Ethiopia found that when children refused to eat, the coping strategies reported by caregivers in questionnaires included increasing food variety and pressure to eat, and additional strategies were observed in videos, including changing caregivers, communicating with the children, and threatening [13]. In this study, a combination of survey and video observation methods was necessary to gain a comprehensive understanding of responsive/nonresponsive feeding practices and to address the gaps in the Chinese literature.

This study had 3 primary aims. First, we sought to provide a quantitative profile of responsive and nonresponsive feeding practices among caregivers of children aged 6‐35 months in China. Every dimension of responsive/nonresponsive feeding was examined. Second, we conducted video observation among a small sample, in order to gain an in-depth understanding of feeding interactions. The video observation allowed us to capture subtle, unreported behaviors and contextual details that surveys might miss, thereby complementing the survey data by providing richness and depth. Third, using the survey data, we aimed to identify key demographic factors (eg, caregiver’s employment status, education level, family income, etc) associated with these feeding practices to inform future interventions. Consequently, this study would contribute to the literature by (1) providing novel and in-depth data on the responsive/nonresponsive feeding practices of Chinese caregivers; (2) demonstrating the value of a mixed methods approach, using a survey method for breadth and an observation method for depth to achieve a more holistic assessment of feeding practices; and (3) identifying potential targets for responsive feeding interventions in China.

## Methods

### Design

This study adopted a combined approach, involving a questionnaire survey conducted among a relatively large sample and a video observation study conducted among a relatively small sample. The survey was reported according to the STROBE (Strengthening the Reporting of Observational Studies in Epidemiology) guidelines, and the video observation was reported in adherence to the COREQ (Consolidated Criteria for Reporting Qualitative Research) checklist.

### Ethical Considerations

Approval to conduct this study was obtained from the Ethics Committee of Peking University (IRB00001052-20047; approval date: August 1, 2020). This study has been performed in accordance with the ethical standards laid down in the 1964 Declaration of Helsinki and its later amendments. All caregivers signed a written informed consent form before any assessment. Adhering to privacy and confidentiality guidelines, all identifiable participant details, including names, hospital numbers, and recognizable features in images, were anonymized or omitted. Participants of the survey received a small toy valued at 5 RMB (US $0.72), while participants of the video observation received 100 RMB (US $14).

### Survey

#### Study Design

A cross-sectional survey was conducted from August to October 2020 in Shijiazhuang City, Hebei Province, China. Methods for participant selection, recruitment, data collection, and the key instrument have been detailed in our previous publication [5,6].

#### Participants

Family members who took the primary responsibility of child caregiving were our potential participants. Caregivers were included if they (1) were permanent residents of the study area; (2) had a certain level of literacy; and (3) had a child at full-term delivery, of normal birth weight, and aged 180 days to 3 years (excluding the full age of 3 years). Caregivers were excluded if they had a child with diseases that affect eating (eg, cold, fever, diarrhea, and food allergies), congenital diseases, infectious diseases, genetic metabolic diseases, deformities, mental diseases (eg, depression), or other serious illnesses (eg, cancer, severe liver or kidney diseases, and AIDS). All included participants signed a written informed consent form before any assessment.

#### Recruitment and Data Collection

This study was advertised through posters at Loudi Township Hospital of Luancheng District. A convenience sampling method was used to recruit caregivers who visited the hospital for routine health check-ups and vaccinations. Eligible caregivers signed an informed consent form after being informed of the purpose and process of the study. Participants were then required to complete a questionnaire via a face-to-face interview with the researcher, which took approximately 20 minutes. They were given a small toy valued at 5 RMB (US $0.72) as an incentive to participate in this study. Researchers checked each completed questionnaire. Participants who provided missing or illogical answers were further contacted for adjustment. Data entry was finally performed, with 10% double entry for quality assurance.

#### Questionnaire

A cross-sectional questionnaire was developed to collect participants’ general demographic characteristics and their responsive/nonresponsive feeding practices. Caregivers’ responsive and nonresponsive feeding practices were measured by a scale, which was based on previous validated scales [16-18] and then further modified by our research team. Details on the construction and validation of this responsive/nonresponsive feeding scale have been provided in our previous publication involving the same study population [5]. In brief, the full Parents’ Feeding Practices Scale for Infant and Young Child (PFPSIYC) was adopted to assess 5 feeding dimensions (including controlling, pressure to eat, the use of food as a reward, emotional feeding, and modeling). The PFPSIYC has been used to assess Chinese caregivers of children aged 6‐23 months, with acceptable reliability and good validity [16]. Some items from the Infant Feeding Style Questionnaire (IFSQ) were used to evaluate caregivers’ responses to dietary cues. The IFSQ has been used among caregivers in the United States, with good reliability [17]. Some items were adopted from the Young Child Feeding Questionnaire (YCFQ) to evaluate active communication and interaction between caregivers. The YCFQ has been applied to caregivers of children aged 6‐18 months in China, with acceptable reliability [18].

To ensure the instrument’s suitability for the context of this study, a pilot study was conducted among 132 caregivers in Hebei Province. This pilot study confirmed the feasibility of the survey procedures and the comprehensibility of the items. The pilot study showed acceptable internal consistency (Cronbach α=0.810) and structural validity (cumulative variance explained=61.6%) of the feeding scale. Following the pilot study, the survey was administered to the population of this study. The structural validity of the scale was good among the current sample, and the structure of each dimension was comparable to the original scales, with eigenvalue >1 for 7 factors (cumulative percentage of variance: 60.388%). The internal reliability was acceptable (Cronbach α: total, 0.765; controlling, 0.740; the use of food as a reward, 0.629; emotional feeding, 0.727; responsiveness to cues, 0.778; active communication and interaction, 0.771; modeling, 0.497; and pressure to eat, 0.441).

In this study, caregivers’ frequency of each feeding practice in the previous 1 month was assessed using a 5-point Likert scale (1=never, 2=rarely, 3=sometimes, 4=often, and 5=always). The score for each feeding dimension was obtained by calculating the mean score of the items within the dimension. To calculate the overall score of responsive feeding, the scorings for nonresponsive feeding practices were first reversed, followed by taking the mean value of all responsive and nonresponsive feeding items. A higher overall score indicated a greater tendency to adopt responsive feeding practices.

#### Data Analysis

Participants’ demographic characteristics and the scores of caregivers’ responsive/nonresponsive feeding practices were described. Frequencies and percentages have been presented for categorical variables. Continuous variables in this study did not meet normal distribution according to a normality test, and thus, they have been presented using medians and quartiles. Mann-Whitney *U* and Kruskal-Wallis tests were used to examine the univariate association between caregivers’ responsive/nonresponsive feeding practices and participants’ demographic characteristics. Finally, multiple linear regression was used to determine the independent effects of participants’ demographic characteristics on caregivers’ responsive/nonresponsive feeding practices. Variables with *P*<.10 in the univariate analysis were entered into the regression model. A variance inflation factor of <5 was used to indicate a low degree of multicollinearity of the independent variables. Statistical analyses were performed using SPSS 22.0 (IBM Corp). A *P* value of <.05 was considered statistically significant.

### Video Observation

#### Study Design and Participants

A video observation study was conducted from August to October 2020 in Luannan County, Tangshan City, Hebei Province, China. Eligible participants were infants and toddlers, and their primary caregivers. The inclusion criteria were the same as those for the survey, and caregivers were required to agree to participate in this observation study. Referring to previous video observation studies on feeding practices [14,19-21] and considering the time, financial, and human resources of this study, 42 caregiver-child pairs were recruited for this study, including 21 pairs each for children aged 6‐23 months and those aged 24‐35 months.

#### Recruitment and Data Collection

Convenience sampling was used to recruit participants. Researchers were led by village committee staff for household visits. Eligible caregivers were informed of the purpose and procedure of the study. Caregivers who agreed to take part in this study signed a written informed consent form and filled in a brief questionnaire on demographic information. The researchers made appointments with the caregivers for the time and place to carry out video recording. The caregivers were required to prepare meals and feed their children in a daily routine manner. On the day of the video recording, the researchers (who played the role of a videographer) dressed in a normal style so as not to arouse the curiosity or anxiety of the children. The researchers recorded the children’s dining occasions of breakfast, lunch, and dinner, according to the instructions of the operation manual (see Multimedia Appendix 1 for details). For younger infants, 2‐3 complementary meals were recorded. The total duration of the videos was about 1.5 hours for each caregiver-child pair. After completing each feeding episode, the researchers performed a quality check (eg, whether the picture was clear and whether the sound was recognizable). If the quality was not up to standard, the corresponding meal was recorded again on a later date until the quality of the video was assured. Upon the completion of all recordings, each caregiver received 100 RMB (US $14) as an incentive. All videos were saved to a password-protected removable hard drive and kept confidential by the researchers.

#### Pilot Study

Three households (one each for children aged <12 months, 12-23 months, and 24‐35 months) were selected to record children’s dining episodes within a day. The pilot study discovered that a meal lasted 5‐30 minutes, and both caregivers and children could easily get used to the recording. The number of feeding episodes recorded (2‐3 times per day) was acceptable to both the participants and researchers. Based on the pilot study, the researchers revised and finalized the operation manual for video recording (Multimedia Appendix 1).

#### Video Coding and Quality Control

Based on the literature, responsive (responsiveness to cues, active communication and interaction, modeling, and creating a good meal environment) and nonresponsive (controlling, the use of food as a reward, emotional feeding, and pressure to eat) feeding practices included 4 dimensions each [3,5,11]. All videos were viewed, and the practices of responsive and nonresponsive feeding were recorded and summarized into a codebook (Multimedia Appendix 2). Notably, the practice of emotional feeding could not be observed in the videos taken, and thus, this was not coded. Next, the number of times each practice appeared was recorded according to the codebook. Taking responsiveness to cues as an example, a caregiver was considered to have this practice as long as any of the corresponding behaviors were shown. A consecutive practice with an interval of <5 seconds was counted as 1, while the same practice with an interval of ≥5 seconds was counted as 2. Coding was performed using the Behavioral Observation Research Interactive Software (BORIS) [22], which automatically summarized the number and duration of each practice in each coded video. Regarding the practice of creating a good meal environment, the researcher scored the overall feeding environment for each feeding episode according to the predefined criteria listed in the codebook. When a criterion was met, a score of 1 was obtained. A higher accumulated score indicated that the caregiver was more likely to create a good meal environment. The scores of creating a good meal environment were recorded in Excel software (Microsoft Corp). All videos were coded by 1 researcher (MA), with 20% of the videos randomly selected for double-coding by another trained coder [23]. The intraclass correlation coefficients (ICCs) between the 2 coders for each feeding practice ranged from 0.587 to 0.885 (*P*<.05), indicating an acceptable level of reliability [24]. Finally, the coding results were processed by counting the number of times the feeding practices occurred at each dining occasion (breakfast/lunch/dinner). The score for creating a good meal environment was also calculated for each dining occasion. The duration of each feeding practice was not analyzed in this study owing to its low ICC values.

Quality control was performed for coding. First, the coders received intensive training on the content of the codebook, the coding process, and the use of coding software before any coding. Second, 20% of double-coding was performed to ensure the reliability of the coding results. Third, each coder signed a data confidentiality agreement before getting access to any data. Video coding was performed on a password-protected computer that was disconnected from the internet during coding. The coding results were kept confidential by the researchers.

#### Data Analysis

First, participants’ demographic characteristics were described, and the chi-square test was used to analyze the difference in demographics between observation and survey participants. Second, the responsive/nonresponsive feeding practices extracted from coding were described, supplementing the results from the survey. Then, for each dining occasion (breakfast/lunch/dinner), the occurrence and observed number of responsive/nonresponsive feeding episodes were presented. The occurrence (%) was calculated as follows: (the number of videos in which responsive/nonresponsive feeding appeared for each occasion/the total number of videos for each occasion) × 100. Since the observed number did not meet a normal distribution, it was described by median (IQR). Statistical analyses were carried out using SPSS 22.0 software, and a *P* value of <.05 was used to indicate statistical significance.

## Results

### General Characteristics of the Participants

Among the 444 participants recruited, 16 did not complete the questionnaire; 2 provided ineligible informed consent; 1 provided a duplicate questionnaire; and 16 had a preterm child, had a child with an abnormal birth weight, or had a child of inappropriate age. Finally, a total of 409 participants were included in the survey. Most caregivers were parents (310/409, 75.8%; including 301 mothers), had a high school education or below (282/409, 68.9%), were unemployed (217/409, 53.1%), and had a normal weight status (207/409, 51.7%). The majority of children were aged 24‐35 months (217/409, 53.1%), were male (214/409, 52.3%), were not the first child in the family (211/409, 51.6%), had a normal weight status (372/409, 93.0%), lived in an extended family (310/409, 75.8%), and had parents with a monthly household income of >5000 RMB (>US $721; 247/409, 60.4%) (Table 1).

**Table 1.** General characteristics of the caregivers and children.

Characteristic	Survey (N=409), n (%)		Video observation (N=42), n (%)		Chi-square (*df*)	*P* value
Caregivers’ characteristics						
Relationship with the child					2.098 (1)	.15
Parent	310 (75.8)		36 (85.7)			
Nonparent	99 (24.2)		6 (14.3)			
Education level					6.749 (1)	.009^a^
High school or below	282 (68.9)		37 (88.1)			
College or above	127 (31.1)		5 (11.9)			
Employment status					2.415 (1)	.12
Employed	192 (46.9)		25 (59.5)			
Unemployed	217 (53.1)		17 (40.5)			
Weight status					0.419 (2)	.81
Underweight	18 (4.5)		1 (2.4)			
Normal weight	207 (51.7)		22 (52.4)			
Overweight or obese	175 (43.8)		19 (45.2)			
Children’s characteristics						
Age (months)					0.143 (1)	.71
6-23	192 (46.9)		21 (50.0)			
24-35	217 (53.1)		21 (50.0)			
Gender					3.152 (1)	.08
Male	214 (52.3)		28 (66.7)			
Female	195 (47.7)		14 (33.3)			
Child order					11.167 (1)	.001^a^
First	198 (48.4)		9 (21.4)			
Second or higher	211 (51.6)		33 (78.6)			
Infant weight status (based on BMI *z* score)					1.478 (2)	.48
Wasting or severe wasting	14 (3.5)		1 (2.4)			
Normal	372 (93.0)		38 (90.5)			
Overweight or obese	14 (3.5)		3 (7.1)			
Family structure					8.722 (1)	.003^a^
Nuclear family	99 (24.2)		19 (45.2)			
Extended family	310 (75.8)		23 (54.8)			
Monthly household income (RMB^b^)					0.502 (1)	.48
≤5000	162 (39.6)		19 (45.2)			
>5000	247 (60.4)		23 (54.8)			

a *P*<.05, chi-square test.

b A currency exchange rate of 1 RMB=US $0.14 is applicable.

A total of 42 caregiver-child pairs were included in the video observation, including 21 pairs each of children aged 6‐23 months and those aged 24‐35 months. In general, the characteristics of the participants in the video observation and survey were similar (Table 1).

### Responsive and Nonresponsive Feeding Practices

The survey measured 7 dimensions of feeding practices. The scores for responsive feeding practices were generally higher than those for nonresponsive feeding practices. The median scores for responsiveness to cues, modeling, and active communication and interaction were 4.00, 3.67, and 3.88, respectively. The median scores for controlling, pressure to eat, the use of food as a reward, and emotional feeding were 3.33, 2.33, 3.00, and 2.67, respectively. The overall responsive feeding score was 3.52 (Table 2).

**Table 2.** Scores of responsive and nonresponsive feeding practices in the survey (N=409).

Behavior	Quartile		
	P_25_^a^	P_50_^b^	P_75_^c^
Nonresponsive feeding practices			
Controlling	2.67	3.33	4.00
Pressure to eat	1.67	2.33	3.00
Use of food as a reward	2.00	3.00	3.50
Emotional feeding	2.00	2.67	3.00
Responsive feeding practices			
Responsiveness to cues	3.67	4.00	4.33
Modeling	3.00	3.67	4.00
Active communication and interaction	3.50	3.88	4.25
Overall score of responsive feeding	3.36	3.52	3.76

a P_25_: 25th percentile.

b P_50_: median.

c P_75_: 75th percentile.

Video observation additionally assessed the dimension of “creating a good meal environment” that could not be examined by the survey. Except for caregivers’ emotional feeding, all dimensions could be observed by the videos (Table 3). A total of 105 videos were recorded, including 27 videos for breakfast, 37 for lunch, and 41 for dinner. In general, the occurrences of responsive feeding practices (75.6%‐97.6%) were higher than those of nonresponsive feeding practices (0%‐46.3%) at main meals. There was little variation in the occurrence of each practice during breakfast, lunch, and dinner. In addition, among nonresponsive feeding, controlling and pressure to eat were more likely to occur than the use of food as a reward. The practice of the use of food as a reward appeared at breakfast and dinner but not at lunch (Table 3). Notably, the practice of the use of food as a reward is very likely to occur during snacking, which was not recorded in this study. Therefore, the occurrence of the use of food as a reward reported here is likely to be underestimated. In terms of the observed number of practices at each dining occasion, the observed number of responsive feeding practices (2-11) was higher than that of nonresponsive feeding practices (0). The score for creating a good meal environment was high at 4 points. For each feeding practice, the observed number and scores at breakfast, lunch, and dinner were similar (Table 4).

**Table 3.** Occurrence of responsive and nonresponsive feeding practices at main meals in the video observation.

Behavior^a^	Occurrence, n (%)		
	Breakfast (n=27^b^)	Lunch (n=37^b^)	Dinner (n=41^b^)
Nonresponsive feeding practices			
Controlling	11 (41)	17 (46)	19 (46)
Pressure to eat	12 (44)	14 (38)	17 (42)
Use of food as a reward	3 (11)	0 (0)	2 (5)
Responsive feeding practices			
Responsiveness to cues	24 (89)	34 (92)	38 (93)
Modeling	24 (89)	31 (84)	31 (76)
Active communication and interaction	26 (96)	35 (95)	40 (98)

a The responsive feeding practice of creating a good meal environment was evaluated according to the score and not occurrence. The nonresponsive feeding practice of emotional feeding could not be observed by video in this study.

b Number of videos.

**Table 4.** Observations of responsive and nonresponsive feeding practices in the videos.

Behavior	Observations, median (IQR)		
	Breakfast (n=27^a^)	Lunch (n=37^a^)	Dinner (n=41^a^)
Nonresponsive feeding practices^b^			
Controlling	0 (0-3)	0 (0-2)	0 (0-2)
Pressure to eat	0 (0-1)	0 (0-2)	0 (0-2)
Use of food as a reward	0 (0-0)	0 (0-0)	0 (0-0)
Responsive feeding practices			
Responsiveness to cues	7 (4-11)	5 (3-13)	6 (3-12)
Modeling	2 (1-4)	2 (1-6)	3 (1-6)
Active communication and interaction	11 (6-17)	10 (5-18)	11 (6-17)
Creating a good meal environment^c^	4 (3-4)	4 (3-4)	4 (3-4)

a Number of videos.

b The nonresponsive feeding practice of emotional feeding could not be observed by video in this study.

c The responsive feeding practice of creating a good meal environment was evaluated by the score and not the number of observations in the videos.

Observations from the videos provided rich and contextual narratives that illustrated responsive/nonresponsive feeding practices. For instance, the practice of “using food as a reward” frequently involved ultraprocessed foods such as sugary drinks and spicy strips. In 1 episode, a mother promised to provide her child with spicy strips for the purpose of encouraging the child’s consumption of the main meal. She said: “OK…spicy strips… If you finish this meal, I will give you spicy strips.*”* Moreover, the practice of “pressure to eat” was observed in the following three dimensions: (1) continue feeding despite the child’s refusal (eg, turning the head away and crying), (2) use distractions (eg, toys and mobile phones) to coax the child into eating, and (3) insist on eating even after satiety cues (eg, verbally expressing fullness and pushing the bowl away). Video descriptions of “responsiveness to cues” have been presented in our previous publication [6].

### Factors Influencing Responsive and Nonresponsive Feeding Practices

The results from univariate analyses that explored factors influencing responsive/nonresponsive feeding practices are shown in Multimedia Appendix 3. After controlling for potential confounders, the results from multivariate analyses are shown in Table 5. For the overall responsive feeding score, caregivers who were nonparents (β=0.13, 95% CI 0.05-0.21) and had a monthly household income of >5000 RMB (>US $721; β=0.07, 95% CI 0.01-0.14) had a higher overall score, while the score was lower among unemployed caregivers (β=−0.07, 95% CI −0.13 to −0.01). For nonresponsive feeding practices, caregivers whose children were not the first child (β=−0.20, 95% CI −0.39 to −0.02) and whose children were aged 24‐35 months (β=−0.20, 95% CI −0.38 to −0.02) had a lower score. The score for pressure to eat was higher among unemployed caregivers (β=0.27, 95% CI 0.11-0.42). The score for the use of food as a reward was lower for caregivers who were nonparents (β=−0.45, 95% CI −0.69 to −0.22). The score for emotional feeding was higher among caregivers whose children were not the first child (β=0.18, 95% CI 0.03-0.34). In terms of responsive feeding practices, the score for responsiveness to cues was higher among caregivers who were nonparents (β=0.43, 95% CI 0.25-0.60), while the score was lower among unemployed caregivers (β=−0.19, 95% CI −0.33 to −0.05). The score for modeling was higher among caregivers whose weight status was underweight (β=0.44, 95% CI 0.07-0.80), and the score was lower among caregivers whose children were aged 24‐35 months (β=0.22, 95% CI 0.07-0.37). Caregivers whose children were not the first child (β=−0.17, 95% CI −0.29 to −0.54) had a lower score for active communication and interaction.

**Table 5.** Multivariate analysis of factors influencing responsive and nonresponsive feeding behaviors assessed in the survey (N=409).

Variable	Nonresponsive feeding practices, β^a^ (95% CI)				Responsive feeding practices, β (95% CI)			Overall score of responsive feeding, β (95% CI)
	Controlling	Pressure to eat	Use of food as a reward	Emotional feeding	Responsiveness to cues	Modeling	Active communication and interaction	
Caregivers’ characteristics								
Relationship with the child: nonparents (vs parents)	—^b^	−0.19 (−0.38 to 0.01)	−0.45 (−0.69 to −0.22)^c^	—	0.43 (0.25 to 0.60)^c^	—	—	0.13 (0.05 to 0.21)^c^
Education level: college or above (vs high school or below)	—	0.16 (−0.02 to 0.34)	0.05 (−0.17 to 0.27)	—	0.03 (−0.13 to 0.19)	—	—	−0.02 (−0.10 to 0.05)
Employment status: unemployed (vs employed)	—	0.27 (0.11 to 0.42)^c^	—	—	−0.19 (−0.33 to −0.05)^c^	—	—	−0.07 (−0.13 to −0.01)^c^
Weight status: underweight (vs normal weight)	—	—	—	—	—	0.44 (0.07 to 0.80)^c^	—	—
Weight status: overweight or obese (vs normal weight)	—	—	—	—	—	0.01 (−0.14 to 0.16)	—	—
Children’s characteristics								
Age: 24-35 months (vs 6-23 months)	−0.20 (−0.38 to −0.02)^c^	—	—	—	0.11 (−0.04 to 0.25)	0.22 (0.07 to 0.37)^c^	—	—
Gender: female (vs male)	—	—	0.16 (−0.03 to 0.34)	—	—	—	—	—
Child order: second or higher (vs first)	−0.20 (−0.39 to −0.02)^c^	—	—	0.18 (0.03 to 0.34)^c^	—	—	−0.17 (−0.29 to −0.54)^c^	−0.06 (−0.12 to 0.01)
Family structure: extended family (vs nuclear family)	—	—	—	—	—	—	−0.06 (−0.20 to 0.08)	—
Monthly household income^d^: >5000 RMB (vs ≤5000 RMB)	—	—	—	—	—	—	—	0.07 (0.01 to 0.14)^c^

a β: unstandardized regression coefficient.

b The variable had a *P* value of >.10 in the univariate analysis (see Multimedia Appendix 3), and it was thus not included in the multivariate analysis.

c *P*<.05, multiple linear regression.

d A currency exchange rate of 1 RMB=US $0.14 is applicable.

## Discussion

### Summary of the Study Findings

This study used a combination of survey and video observation methods to obtain a comprehensive understanding of the current status of caregivers’ responsive and nonresponsive feeding practices among children aged 6‐35 months and performed multivariate analyses of the survey data to explore factors influencing responsive and nonresponsive feeding practices. Both survey and video observation methods found that the frequencies of responsive feeding practices were higher than those of nonresponsive feeding practices. Video observation revealed that there was little variation in the occurrence/score of each feeding practice in main meals (ie, breakfast, lunch, and dinner). The survey demonstrated that caregivers’ relationships with their children, employment status, and weight status; children’s age and order; and the monthly household income were factors influencing responsive/nonresponsive feeding practices.

### Caregivers’ Responsive and Nonresponsive Feeding Practices

Both survey and video observation methods in this study found that caregivers adopted responsive feeding practices more frequently than nonresponsive feeding practices. In our survey, the scores for nonresponsive feeding practices were at a moderate to low level, similar to findings reported in other Chinese studies [16,25]. For example, a study in Hong Kong revealed that the scores for the use of food as a reward and emotional feeding were 2.68 (SD 0.66) and 2.31 (SD 0.70) points, respectively [26]. However, the score for pressure to eat among caregivers of children aged 3‐18 months in Spain was 3.46 (SD 0.82) points [27], which is higher than that in our study. The scores for responsive feeding practices in our study were moderately high and were consistent with the findings of other national and international studies. For instance, a study in the United States revealed that the score for responsiveness to cues among children aged 3‐18 months was 4.49 (SD 0.04) points [28]. The median scores for behaviors related to active communication and interaction among caregivers whose children were aged 18 months in Shanghai were between 4 and 5 points, indicating a relatively high level [18].

The video observation confirmed the results of the survey. In daily meals, caregivers adopted responsive feeding practices more frequently than nonresponsive feeding practices. Caregivers likely prepared a variety of nutritious foods for their children. Responsive feeding practices, such as modeling, are effective strategies for promoting children’s intake of these healthy foods [6]. Furthermore, video observation found that the number of responsive feeding observations was similar among breakfast, lunch, and dinner. Similarly, a video observation study among children aged 12‐36 months in the United States indicated that caregivers’ feeding practices during each main meal had a certain degree of stability. Observation of a single main meal might be an alternative approach to examine responsive feeding in future studies, if resources are limited.

### Factors Influencing Caregivers’ Responsive and Nonresponsive Feeding Practices

Previous studies on feeding practices were mainly conducted among mothers. Our study adds to the literature by examining responsive and nonresponsive feeding practices among both parents and nonparents. In this study, the scores of overall responsive feeding and the practice of responsiveness to cues among nonparents (99% grandparents) were higher than those among parents. However, the score of the use of food as a reward among nonparents was lower than that among parents. This might be because grandparents were patient and responsible when feeding their grandchildren and had sufficient time to adopt responsive feeding strategies [29,30]. From our video observation, grandparents waited for a while after each feed and did not provide the next feed until the child swallowed the food (data not shown). Moreover, grandparents had previous experience in feeding children. Finally, with the broad use of the internet and smartphones, grandparents were knowledgeable in using appropriate feeding strategies. Our results imply that education on optimal feeding practices should be given to parents. For instance, local maternal and child health centers might deliver responsive feeding guidance (eg, recognizing children’s satiety cues and optimizing mealtime interactions) to parents during children’s routine health check-ups and vaccination visits. On the contrary, some studies failed to report differences in pressure to eat and the use of food as a reward between grandparents and parents, and some studies found that grandparents were more inclined to adopt the pressure to eat practice than parents [29,31]. Children in these studies were older than our study participants [31], which might explain the variation in results between our study and previous studies.

In this study, compared with employed caregivers, unemployed caregivers scored lower in overall responsive feeding and responsiveness to cues but scored higher in pressure to eat. Moreover, the score of overall responsive feeding was lower among caregivers whose monthly household income was ≤5000 RMB (≤US $721). Caregivers with a lower socioeconomic status likely paid less attention to responsive feeding practices. Likewise, a qualitative study in Thailand among factory worker parents of preschool children demonstrated that family income was one of the most influential factors on children’s food choice, as parents prioritized their budget on food over nutritional quality due to economic constraints [32]. Therefore, interventions to enhance responsive feeding in China should attempt to target low-income groups. For example, community health centers might consider offering feeding workshops aligned with intergenerational care norms free of charge for low-income groups. Free personalized consultation could also be provided by community health care workers.

In this study, caregivers of children aged 24‐35 months had a lower score for controlling and a higher score for modeling, in comparison with caregivers of children aged 6‐23 months. It is likely that older children could express their dietary signals well, making it easier for caregivers to respond more appropriately. With an increase in their independence, children are more likely to eat on their own [33]. Caregivers’ use of the modeling strategy could help the development of children’s healthy eating habits. Therefore, specific education on feeding practices should be delivered to caregivers by considering the age of their children. Caregivers of younger children (less than 24 months of age) urgently need responsive feeding education.

Caregivers whose children were not the first child in the family were more likely to use emotional feeding but less likely to use active communication and interaction, and controlling strategies, in comparison with caregivers whose children were the first child. Based on the theory of family systems, the dilution of resources (time, energy, etc) for raising multiple children may affect caregivers’ feeding practices [34]. With an increase in the number of children in the family, caregivers’ time and energy allocated to each child decrease. Therefore, the frequency of caregiver-child interaction and verbal communication during feeding decreases [34]. Caregivers’ practice of controlling children’s eating also decreases. Caregivers are more likely to become impatient and use emotional feeding. However, a cross-sectional study in Thailand reported that the first child was more likely to receive inappropriate complementary feeding [35]. The Thai study focused on children’s dietary intake and diversity, while our study focused on the behaviors of the caregiver-child interaction. Differences in research focus might account for the variations in findings between the studies.

Compared with caregivers having a normal weight status, underweight caregivers in this study adopted more modeling behaviors. The reason may be that underweight caregivers are more concerned about insufficient dietary intake or being underweight in childhood, and thus, they play the role of an eating model for their children and ensure that their children have a good nutritional status.

Notably, there was no difference in responsive/nonresponsive feeding practices when considering the child’s gender. It appears that Chinese traditional patriarchal attitudes did not influence caregivers’ feeding practices among our study participants. Additionally, caregivers’ education, children’s weight status, and the family structure were not associated with caregivers’ responsive/nonresponsive feeding practices in this study.

### Strengths and Limitations

This study adopted a combined approach involving survey and video observation methods to investigate responsive/nonresponsive feeding practices among caregivers of children aged 6‐35 months. This approach provided an objective and comprehensive assessment of responsive/nonresponsive feeding practices. Findings from the survey and observation assessments were confirmatory and complementary to each other. The questionnaire had good reliability and validity and could comprehensively measure all dimensions of responsive and nonresponsive feeding practices. Video observation could objectively capture the actual feeding environment and caregiver-child interaction and assess the practice of “creating a good meal environment,” which could not be measured by questionnaires. Moreover, this study used multivariate analysis to explore the factors influencing responsive and nonresponsive feeding practices. The findings of this study would be useful in the development of interventions and strategies to promote responsive feeding and in the identification of targeted populations for interventions.

The limitations of this study should be acknowledged. First, this study was conducted in some areas of Hebei Province. The results from this study might not be representative of caregivers living in other areas. Moreover, the caregivers in our survey sample had a higher education level but a lower employment rate than the general population [36]. National data indicate that the population in Hebei Province is at the top level and its economic output (reflected by gross domestic product) is at the middle level in China [36]. The health literacy of residents in Hebei Province [37] was similar to that at the national level (27.78%), according to 2022 surveillance data [38], implying that our findings might represent the Chinese population to some extent. Second, the use of food as a reward is more likely to occur in snacking occasions than during main meals. However, our video observation only recorded feeding episodes during main meals. Thus, the occurrence of the use of food as a reward reported in our video observation might be lower than the actual status. Third, owing to the COVID-19 epidemic, video observation could not be carried out at the planned location (Shijiazhuang city, where the survey was conducted). After careful discussion by the research team, Tangshan city was selected as an alternative location. The 2 cities are located in the same province, with a distance of 420 kilometers, ensuring the homogeneity of the samples to some extent. Fourth, the Hawthorne effect may have existed in the video recording process owing to the presence of the camera or videographer. Nevertheless, a number of efforts were taken to minimize the Hawthorne effect. For instance, we conducted the recording in a familiar environment (ie, home); we explicitly instructed caregivers to behave as usual prior to each recording; and the videographer dressed normally, remained silent, stayed hidden in a corner, and avoided participating in any feeding practices during the recording.

### Conclusion

With a combined approach involving survey and video observation methods, this study revealed that the frequency of responsive feeding practices was higher than that of nonresponsive feeding practices among Chinese caregivers whose children were aged 6‐35 months. The combined approach might serve as a methodological reference for research on feeding practices. Moreover, the survey demonstrated that parental, unemployed, and low-income caregivers were less likely to use responsive feeding strategies. Health care providers should consider this finding and target such groups for feeding education and intervention.

## Supplementary material

10.2196/78028 Multimedia Appendix 1 Operation manual for video recording.

10.2196/78028 Multimedia Appendix 2 Codebook of responsive and nonresponsive feeding practices.

10.2196/78028 Multimedia Appendix 3 Scores of responsive and nonresponsive feeding practices categorized by different demographic characteristics in the survey.

## Abbreviations

COREQ: Consolidated Criteria for Reporting Qualitative Research
ICC: intraclass correlation coefficient
IFSQ: Infant Feeding Style Questionnaire
PFPSIYC: Parents’ Feeding Practices Scale for Infant and Young Child
STROBE: Strengthening the Reporting of Observational Studies in Epidemiology
WHO: World Health Organization
YCFQ: Young Child Feeding Questionnaire
