# Supplementary material for: Responsive Feeding Practices Among Caregivers of Children Aged 6-35 Months in China: Descriptive Study Involving Survey and Video Observation Methods
Source: JMIR Pediatr Parent. 2026 Feb 26;9:e78028. doi: 10.2196/78028 (PMC12945353; doi:10.2196/78028)
Supplement: Multimedia Appendix 1 [file pediatrics-v9-e78028-s001.docx]

**Multimedia Appendix 1.** Operation manual for video recording.

- Put the video recorder onto the tripod. Adjust the height of the tripod, so that the infant, caregiver and the foods on the table can be recorded.
- Record a testing video, in order to check a) whether the infant, caregiver, and foods are recorded; b) whether the facial expressions, behaviors, and movements of the infant and caregiver are clearly captured; and c) whether the voices of infant and caregiver are clearly heard.
- Formal recording begins when the infant sits at the dining table and is ready to eat. Recording ends when the infant finishes eating and leaves the table or when the foods are removed.
- During the recording process, if situation unrelated to feeding (e.g. infant urinating) occurs, the recording should be paused and be continued when the infant continues to eat.
- During the recording process, if the infant leaves the dining table and the caregiver follows to feed him/her, the video recorder should be adjusted to capture these movement.
- During the recording process, the cameraman should keep silent and not involve in the feeding process; the video recorder should be located in a corner so that it is not easily noticed by the infants; and the cameraman should check whether the video recorder is working appropriately when necessary.
- Upon the completion of the recording, the researcher reviews the video and performs quality checking (e.g. whether the picture is clear, whether the sound can easily be recognized, etc.). If the video does not pass quality checking, it should be deleted permanently. Further recordings should be conducted in another days until the quality requirements are met.
- Upon the completion of one day’s recording, the videos should be saved in a password-protected portable hard drive which is kept by the researcher.
