# Supplementary material for: Responsive Feeding Practices Among Caregivers of Children Aged 6-35 Months in China: Descriptive Study Involving Survey and Video Observation Methods
Source: JMIR Pediatr Parent. 2026 Feb 26;9:e78028. doi: 10.2196/78028 (PMC12945353; doi:10.2196/78028)
Supplement: Multimedia Appendix 2 [file pediatrics-v9-e78028-s002.docx]

**Multimedia Appendix 2.** Codebook of responsive and nonresponsive feeding practices.^§^

| **Behaviors** | **Description** |
| --- | --- |
| **Controlling** | -The caregiver limits the amount of food the child can eat. (The caregiver stops feeding after emptying the bowl, even the child is not yet full).  -The caregiver controls the type of food the child eats. (When the child wants to eat meat, the caregiver does not provide him/her. The caregiver feeds the child carrot that the child does not want, and makes several attempts to coax the child to eat).  -The caregiver controls the child’s eating speed. (The caregiver urges the child to eat quickly when chewing. The caregiver encourages the child to compete with other children in fast eating). |
| **Pressure to eat** | -The caregiver continues feeding the child even at food refusal.  -When the child refuses to eat, the caregiver uses some strategies [e.g. playing their phones, toys or spoons, using language stimulation (“hit you”, “take you to the playground”, “find someone to play with you”), using body control, chasing the child to feed, changing caregivers] to encourage the child to eat more.  -When the child is full, the caregiver still forces he/she to empty the plate. |
| **Food reward** | - To make the child perform well, the caregiver uses food (e.g. lollipops, spicy snack) as a reward or punishment |
| **Responsiveness to cues** |  |
| **a. Responsiveness to satiety cues** | -The caregiver perceives that the child is full and asks accordingly.  -When the child shows that he/she is full, the caregiver confirms with the child and stops feeding him/her. |
| **b. Responsiveness to the cues during the feeding process** | -When the child shows that the food is hot, the caregiver tries to cool the food (e.g. blowing, cutting the food into small pieces, stirring the food).  -The caregiver observes/asks whether the child has swallowed the food, and does not introduce another bite until the food is fully swallowed.  -The caregiver discusses with the child which food he/she wants to eat.  -When the child wants food, the caregiver offers him/her promptly.  -When the child spits out the food, the caregiver catches the food immediately.  -If the child refuses to eat, the caregiver takes the food away.  -When the child chokes, the caregiver lightly taps his/her back.  -When the child finishes eating a bite of food, the caregiver responds to the child verbally, such as “really clever”, “eating bun”, “taste good?”.  -The caregiver perceives that the child is thirsty and asks accordingly, or offers the child water. |
| **Modeling** | - The caregiver teaches the child how to eat. (The caregiver asks the child to “open their mouths” or say “ah”. The caregiver demonstrates opening mouth to the child).  - The caregiver teaches the child about good eating behaviors. For elder child, the caregiver tells he/she not to use hands, and use chopsticks or spoons instead. The caregiver tells the child how to use tableware appropriately. The caregiver tells the child not to touch his/her mouth. The caregiver tells the child not to taste his/her hands. The caregiver tells the child to eat staple food, meat and vegetables together. The caregiver tells the child to sit down and eat. The caregiver tells the child to swallow the last mouthful of food before eating /drinking. The caregiver tells the child not to eat while going to bed. The caregiver tells the child to eat slowly. The caregiver tells the child how to pick up smaller dishes. The caregiver tells the child not to eat lollipops until finishing main meal and explains the reasons. The caregiver tells the child to leave the table when they are full. The caregiver tells the child to “eat well” and sit well. The caregiver tells the child not to consume mantou with a spoon, and to bite it instead. The caregiver asks the child to wash hands before having meals).  - The caregiver demonstrates her own good eating behaviors. (The caregiver eats with the child and has good eating behavior.) |
| **Active communication and interaction** | - (Language) When the child speaks, the caregiver responds to him/her.  - (Language) When the child is distracted, the caregiver verbally attracts his/her attention to food, such as “come here, let's have some food”.  - (Expression) When the child looks at the caregiver, the caregiver then looks at the child.  - (Expression) When the child smiles at the caregiver, the caregiver then smiles at the child.  - (Action) The caregiver feeds the child face-to-face.  - (Action) When the child wants to touch or hug his/her caregiver, the caregiver allows.  - (Action) The caregiver plays with the child (e.g. clapping).  - (Action) The caregiver wipes the child's hands, mouth or face.  - (Action) The child hands some items to the caregiver (e.g. paper, food) and the caregiver takes them. |
| **Creating a good meal environment^** | - The environment is spacious and bright.  - No interference factors (e.g. television and mobile phone are off, no other people chatting, no siblings disturbing).  - The child is seated comfortably. (The caregiver provides or replaces comfortable dining chair for the child. The caregiver adjusts the child’s seating position if he/she has a tendency to slide at dinning).  - The type and size of the tableware are suitable for the child. (Bowls should not be too large for the child to eat on their own. Cute shaped plates are used for the child.) |

^§^The practices of “emotional feeding” could not be observed in our videos, and were thus not coded.

^For the practice of “creating a good meal environment”, four predefined criteria are listed. When a criteria is met, one score is obtained. The higher the accumulated score, the caregiver is more likely to create a good meal environment.
