# Supplementary material for: Responsive Feeding Practices Among Caregivers of Children Aged 6-35 Months in China: Descriptive Study Involving Survey and Video Observation Methods
Source: JMIR Pediatr Parent. 2026 Feb 26;9:e78028. doi: 10.2196/78028 (PMC12945353; doi:10.2196/78028)
Supplement: Multimedia Appendix 3 [file pediatrics-v9-e78028-s003.docx]

**Multimedia Appendix 3.** Scores of responsive and nonresponsive feeding practices categorized by different demographic characteristics in the survey.^§^ (n = 409)

| Variable | Categories | Non-responsive feeding practices score, P_50_ (P_25_, P_75_) | | | |  | Responsive feeding practices score, P_50_ (P_25_, P_75_) | | | The overall score of responsive feeding, P_50_ (P_25_, P_75_) |
| --- | --- | --- | --- | --- | --- | --- | --- | --- | --- | --- |
|  |  | Controlling | Pressure to eat | Food reward | Emotional feeding |  | Responsiveness to cues | Modeling | Active communication and interaction |  |
| ***Caregivers’ characteristics*** | |  |  |  |  |  |  |  |  |  |
| Relationship to the child | Parents | 3.33 (2.67, 4.00) | 2.33 (2.00, 3.00) ^**^ | 3.00 (2.50, 3.50) ^**^ | 2.67 (2.33, 3.00) |  | 4.00(3.33, 4.33) ^**^ | 3.67(3.00, 4.00) | 3.88(3.50, 4.25) | 3.52 (3.32, 3.72) ^* *^ |
|  | Non- parents | 3.67 (2.67, 4.00) | 2.33 (1.67, 3.00) | 2.50 (1.00, 4.00) | 2.67 (1.67, 3.33) |  | 4.00(4.00, 5.00) | 3.67(3.33, 4.00) | 3.88(3.50, 4.38) | 3.60 (3.40, 3.92) |
| Education level | High school or below | 3.33 (2.67, 4.00) | 2.33 (1.67, 3.00) ^**^ | 3.00 (2.00, 3.50)^*^ | 3.67 (2.00, 3.33) |  | 4.00 (3.67, 4.67)^**^ | 3.67(3.00,4.00) | 3.88(3.38, 4.25) | 3.52 (3.36, 3.80) ^*^ |
|  | College or above | 3.33 (3.00, 4.00) | 2.33 (2.00, 3.00) | 3.00 (2.50, 3.50) | 2.67 (2.33, 3.00) |  | 4.00 (3.33, 4.33) | 3.67 (3.33, 4.00) | 3.88 (3.63, 4.38) | 3.48 (3.32, 3.72) |
| Employment status | Employed | 3.33 (2.67, 4.00) | 2.33 (1.67, 2.67) ^**^ | 3.00(2.00, 3.50) | 2.67(2.00, 3.00) |  | 4.00(3.67, 4.67) ^**^ | 3.67(3.00, 4.00) | 3.88(3.63, 4.38) | 3.56 (3.34, 3.80) ^**^ |
|  | Unemployed | 3.33 (2.67, 4.00) | 2.67 (2.00, 3.00) | 3.00(2.50, 3.50) | 2.67(2.33, 3.33) |  | 4.00(3.67, 4.33) | 3.67(3.33, 4.00) | 3.88(3.50, 4.25) | 3.48 (3.32, 3.68) |
| Weight status | Underweight | 3.33 (2.00, 4.00) | 2.17 (1.92, 3.67) | 3.00(2.50, 4.00) | 2.67(2.25, 3.42) |  | 4.00(3.58, 4.08) | 4.00(3.67, 4.08) ^*^ | 4.00(3.88, 4.53) | 3.60 (3.47, 3.82) |
|  | Normal | 3.33 (3.00, 4.00) | 2.33 (1.67, 3.00) | 3.00(2.50, 3.50) | 2.67(2.00, 3.00) |  | 4.00(3.67, 4.33) | 3.67(3.00, 4.00) | 3.88(3.50, 4.38) | 3.52 (3.32, 3.76) |
|  | Overweight or obese | 3.33 (2.67, 4.00) | 2.33 (2.00, 3.00) | 3.00(2.00, 3.50) | 2.67(2.00, 3.33) |  | 4.00(3.67, 4.33) | 3.67(3.00, 4.00) | 3.88(3.50, 4.25) | 3.52 (3.32, 3.76) |
| ***Children’ s characteristics*** | |  |  |  |  |  |  |  |  |  |
| Age (months) | 6-23 | 3.50 (3.00, 4.00) ^**^ | 2.33(1.67, 3.00) | 3.00(2.00,3.50) | 2.67(2.00,3.00) |  | 4.00(3.67, 4.33) ^**^ | 3.67(3.00, 4.00)^**^ | 4.00(3.63, 4.38) | 3.54 (3.36, 3.76) |
|  | 24-35 | 3.33 (2.67, 4.00) | 2.33(2.00,3.00) | 3.00(2.50,3.50) | 2.67(2.00,3.33) |  | 4.00 (3.67, 4.67) | 3.67(3.33, 4.00) | 3.88(3.38,4.12) | 3.52 (3.32, 3.76) |
| Gender | Male | 3.33 (2.67, 4.00) | 2.33(1.67, 3.00) | 3.00(2.00, 3.50) ^*^ | 2.67(2.00, 3.00) |  | 4.00(3.58, 4.33) | 3.67(3.00, 4.00) | 3.88(3.50, 4.25) | 3.52 (3.32, 3.80) |
|  | Female | 3.33 (2.67, 4.00) | 2.33(2.00, 3.00) | 3.00(2.50, 3.50) | 2.67(2.33, 3.33) |  | 4.00(3.67, 4.33) | 3.67(3.00, 4.00) | 3.88(3.50, 4.38) | 3.52 (3.36, 3.76) |
| Child order | 1 | 3.67(3.00, 4.00) ^* *^ | 2.33(1.92, 3.00) | 3.00(2.00, 3.50) | 2.67(2.00, 3.00)^**^ |  | 4.00(3.67, 4.42) | 3.67(3.33, 4.00) | 3.88(3.63, 4.50) ^* *^ | 3.56 (3.36, 3.80) ^* *^ |
|  | >=2 | 3.33(2.67, 4.00) | 2.33(1.67, 3.00) | 3.00(2.50, 3.50) | 2.67(2.33, 3.33) |  | 4.00(3.67, 4.33) | 3.67(3.00, 4.00) | 3.88(3.38, 4.13) | 3.48 (3.32, 3.72) |
| Family structure | Nuclear family | 3.33(2.67, 4.00) | 2.33(1.67, 3.00) | 3.00(2.50, 4.00) | 2.67(2.00, 3.00) |  | 4.00(3.67, 4.67) | 3.67(3.33, 4.00) | 3.88(3.50, 4.25) ^* *^ | 3.52 (3.32, 3.76) |
|  | Extended family | 3.33(2.67, 4.00) | 2.33(1.67,3.00) | 3.00(2.00,3.50) | 2.67(2.00, 3.08) |  | 4.00(3.67, 4.33) | 3.67(3.00, 4.00) | 3.88(3.50, 4.28) | 3.52 (3.36, 3.76) |
| Monthly household income (RMB) | ≤5000 | 3.33(2.67, 4.00) | 2.33(1.67, 3.00) | 3.00(2.50, 3.50) | 2.67(2.33, 3.08) |  | 4.00(3.67, 4.33) | 3.67(3.00, 4.00) | 3.75(3.38, 4.13) | 3.52 (3.32, 3.72) ^**^ |
|  | >5000 | 3.33(2.67, 4.00) | 2.33(2.00, 3.00) | 3.00(2.00, 3.50) | 2.67(2.00, 3.00) |  | 4.00(3.67, 4.67) | 3.67(3.33, 4.00) | 3.88(3.50, 4.38) | 3.52 (3.36, 3.80) |
| Infant weight status (based on BMIZ) | Wasting or severe wasting | 3.33(3.00, 4.00) | 3.00(2.00, 3.50) | 3.00(2.25, 3.76) | 2.67(2.17, 3.67) |  | 4.00(3.33, 4.50) | 3.67(3.00, 4.00) | 3.88(3.44, 4.31) | 3.52 (3.26, 3.76) |
|  | Normal | 3.33(2.67, 4.00) | 2.33(1.67, 3.00) | 3.00(2.00, 3.50) | 2.67(2.00, 3.00) |  | 4.00(3.67, 4.33) | 3.67(3.00, 4.00) | 3.88(3.50, 4.25) | 3.52 (3.36, 3.80) |
|  | Overweight or obese | 4.00(3.42, 4.50) | 2.17(1.67, 3.00) | 2.75(2.13, 3.50) | 2.67(2.33, 3.00) |  | 4.00(3.33, 4.58) | 3.67(3.42, 4.00) | 4.00(3.69, 4.47) | 3.50 (3.40, 3.74) |

P50: the median, P25: the 25th percentile, P75: the 75th percentile.

§Comparison of the differences in medians across demographic subgroups, Mann-Whitney U or Kruskal-Wallis test. *P<0.10, **P<0.05.
